# *In Vitro* Selection of a Single-Stranded DNA Molecular Recognition Element against Atrazine

**DOI:** 10.3390/ijms150814332

**Published:** 2014-08-18

**Authors:** Ryan M. Williams, Cassandra L. Crihfield, Srikanth Gattu, Lisa A. Holland, Letha J. Sooter

**Affiliations:** 1 Department of Pharmaceutical Sciences, West Virginia University, 1 Medical Center Drive, PO Box 9530, Morgantown, WV 26506, USA; E-Mail: rwilliams@mix.wvu.edu; 2 C. Eugene Bennett Department of Chemistry, West Virginia University, Morgantown, WV 26506, USA; E-Mails: clcrihfield@mix.wvu.edu (C.L.C.); srgattu@mix.wvu.edu (S.G.); lisa.holland@mail.wvu.edu (L.A.H.)

**Keywords:** SELEX, *in vitro* selection, aptamer, molecular recognition element (MRE), atrazine

## Abstract

Widespread use of the chlorotriazine herbicide, atrazine, has led to serious environmental and human health consequences. Current methods of detecting atrazine contamination are neither rapid nor cost-effective. In this work, atrazine-specific single-stranded DNA (ssDNA) molecular recognition elements (MRE) were isolated. We utilized a stringent Systematic Evolution of Ligands by Exponential Enrichment (SELEX) methodology that placed the greatest emphasis on what the MRE should not bind to. After twelve rounds of SELEX, an atrazine-specific MRE with high affinity was obtained. The equilibrium dissociation constant (K_d_) of the ssDNA sequence is 0.62 ± 0.21 nM. It also has significant selectivity for atrazine over atrazine metabolites and other pesticides found in environmentally similar locations and concentrations. Furthermore, we have detected environmentally relevant atrazine concentrations in river water using this MRE. The strong affinity and selectivity of the selected atrazine-specific ssDNA validated the stringent SELEX methodology and identified a MRE that will be useful for rapid atrazine detection in environmental samples.

## 1. Introduction

Atrazine is a chlorotriazine herbicide used widely in crop production. An estimated 76.5 million pounds are used per year in the United States alone [1]. Its use is estimated to increase crop yields 4%–6% [2]. The economic benefits of atrazine use have led to its continued application for many agricultural processes.

The widespread application of atrazine, however, leads to contamination of aquatic ecosystems and water supplies. Dependent upon rainfall, up to 20% of atrazine applied to crops may run off or be leached from soils [3]. Streams bordering croplands applying atrazine have been shown to be contaminated at levels up to 1000 μg·L^−1^. Additionally, some drinking water reservoirs in the U.S. have up to 88.4 μg·L^−1^. Removal of atrazine in water treatment plants is possible, but varies widely throughout the US [4]. The US Environmental Protection Agency (EPA) Maximum Contaminant Level (MCL) is 3 μg·L^−1^ for human exposure to atrazine [5]. The European Union has banned use of atrazine due to hazards and contamination levels above recommended limits [6].

The recommended limit of exposure is based on the potential negative effects of atrazine. Atrazine exposure at environmentally relevant levels affects aquatic organisms; including feminization of male frogs [7], salamander desiccation [8], and endocrine disruption in fish [9]. Endocrine disruption is also a potential mechanism of atrazine action in humans and other mammals [10,11,12]. It has been shown to interact with the steroidogenic transcription factor SF1 and G protein-coupled estrogen receptor 1 (or GPCR30), causing signaling pathways to be upregulated. This may lead to hormonally-linked cancers and reproductive abnormalities. Additionally, atrazine may affect innate immune function and increase damage caused by other toxins such as arsenic [13,14]. There is also evidence that contradicts an EPA finding that atrazine is not a potential carcinogen [15]. It is therefore of critical importance that food and water supplies be routinely monitored for atrazine levels.

The current methods of atrazine detection are either time- and labor-intensive or are not specific for the herbicide. The most common method is gas chromatography, which requires extensive sample preparation [16]. Combinatory chromatographic methods have been used to determine the presence of atrazine in both serum and urine [17,18]. Antibody-based assays are also used, however the antibody used cannot differentiate between atrazine and closely-related molecules [19,20].There also has been a DNA binding element previously selected for atrazine which had a dissociation constant of 890 nM [21]. This affinity, is much less sensitive than necessary for the desired sensing application [22,23]. Furthermore, this binding element was only tested for cross-reactivity against one closely-related structure, and it was not significantly selective below the micromolar range. It is therefore necessary to identify an easily-synthesized binding element with high affinity and selectivity which may be incorporated into rapid sensing devices. Utilizing SELEX (Systematic Evolution of Ligands by Exponential Enrichment) an atrazine-specific Molecular Recognition Element (MRE) was identified with sub-nanomolar affinity.

Molecular Recognition Elements are biological molecules that strongly and selectively bind to a target of interest. They are produced by the *in vitro* selection process initially described by the Gold laboratory in 1990 [24]. Nucleic acid MREs are typically selected from a large (~10^15^) library of single-stranded DNA or RNA molecules. The library is iteratively enriched for molecules which bind to the target of interest. The selected MRE may then be used for applications such as detection of the target molecule.

A single-stranded DNA (ssDNA) MRE with high affinity and specificity for atrazine was isolated using a stringent SELEX scheme. This work enriched the library for the target of selection but also focused on multiple stringent negative selections against undesirable binding targets. Those negative targets were chosen by chemical similarities, such as target metabolites, or the environmental proximity to which they are found with the target. The selection scheme for atrazine was designed to ensure the MRE would bind to neither atrazine metabolites diamino-chloro-triazine (DACT) or desethyl atrazine nor the closely-related herbicide simazine [25]. It was also designed so that the MRE would not bind to propanil, 2,4-D acid, or malathion, which are pesticides found at similar levels and environments to atrazine [26,27,28,29]. Additionally, we have utilized this MRE to detect atrazine spiked into an environmental sample. The selected MRE thus will be useful in a sensing device, allowing for specific atrazine detection (e.g., [30,31,32]).

## 2. Results and Discussion

### 2.1. Selection of Atrazine-Specific MREs

**Figure 1:**
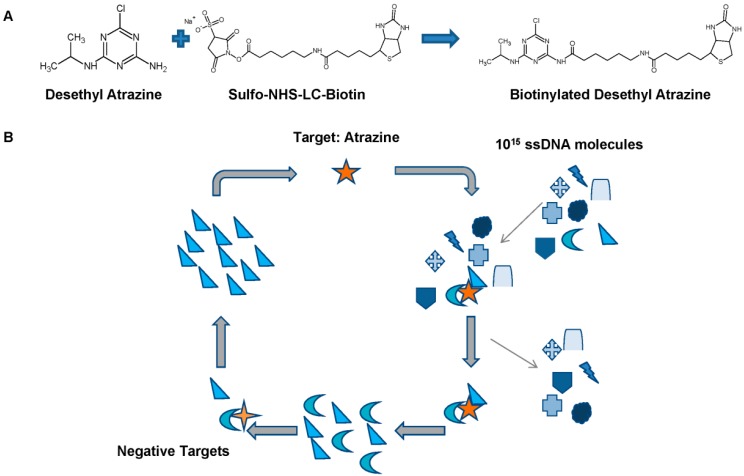
(**A**) Scheme showing the biotinylation of desethyl atrazine for magnetic bead immobilization; (**B**) Scheme depicting the SELEX process. A library of 10^15^ ssDNA molecules are incubated with the target atrazine. Those that bind are amplified and incubated with multiple negative targets. Those that do not bind the negative targets are amplified and subjected to additional rounds of *in vitro* selection.

**Figure 2:**
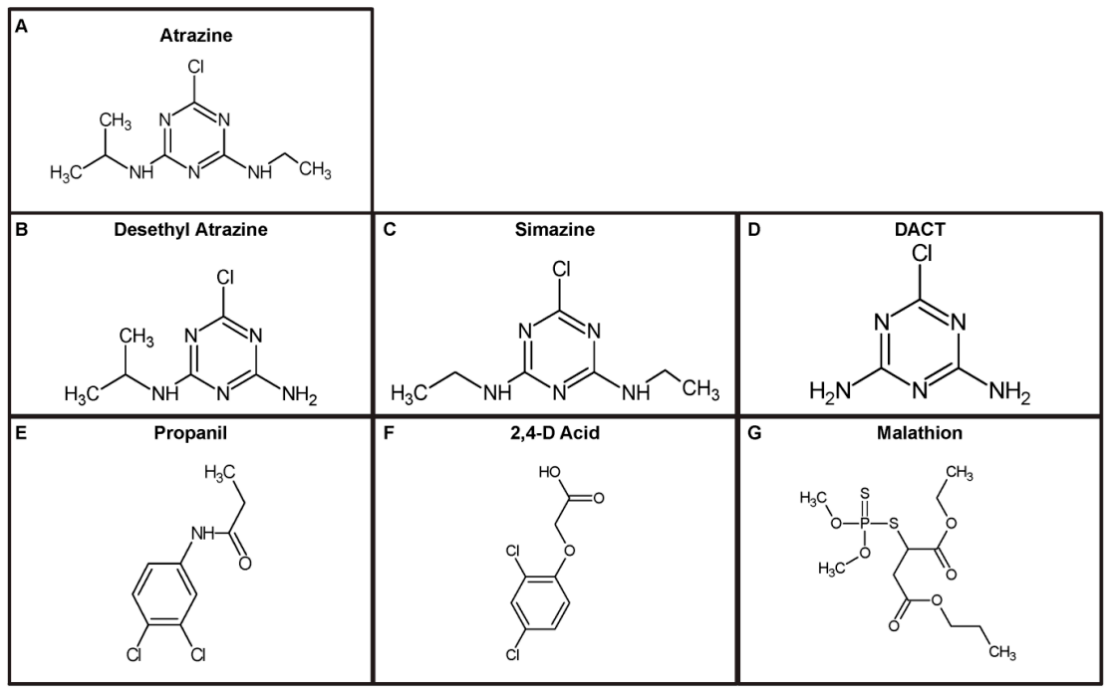
Chemical structures of molecules used in the SELEX scheme and cross-binding assays. (**A**) Structure of the herbicide and target of selection atrazine; (**B**) Structure of atrazine derivative desethyl atrazine, used to immobilize onto magnetic beads; (**C**,**D**) Structures of simazine and diamino-chloro-triazine (DACT): chemically similar to atrazine, and used in rounds of negative selection; (**E**–**G**) Structures of propanil, 2,4-D acid, and malathion: pesticides found in ecologically relevant environments to atrazine and used in negative selections.

**Figure 3:**
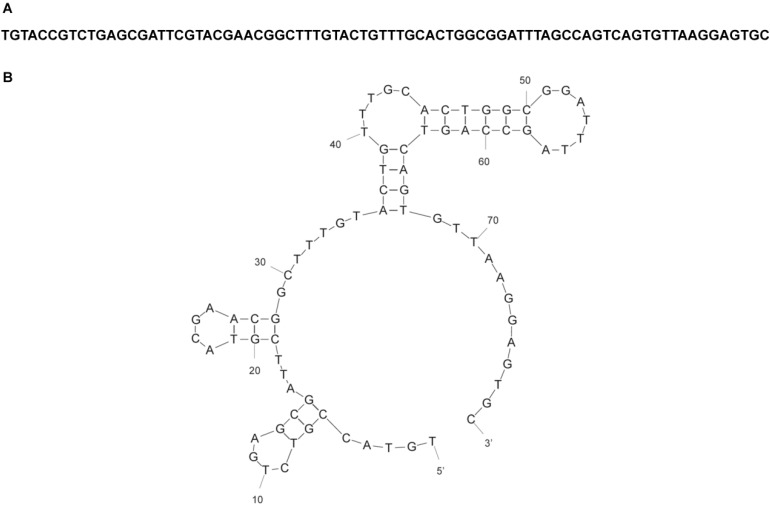
Sequence and structure of R12.23 ssDNA MRE. (**A**) ssDNA sequence of atrazine MRE R12.23; (**B**) Predicted R12.23 secondary structure by Mfold [33].

Twelve rounds of SELEX were completed to obtain ssDNA MREs which specifically bind to atrazine (Table 1, Figure 1). The selection scheme was designed so that the ssDNA MREs will bind to atrazine in solution and bind preferentially over the immobilization substrate and malathion, propanil, 2,4-D acid, DACT, or simazine (Figure 2). After every third round of selection (rounds 3, 6, 9, 12), 30–50 random sequences of the ssDNA molecules in the library were obtained for consensus sequence family analysis. From the Round 12 library, three sequences were chosen based on their inclusion in consensus sequence families and predicted tertiary structures (Table 2). Those sequences, R12.23, R12.28, and R12.57 were assayed for their binding affinity to atrazine. Two of those sequences, R12.28 and R12.57 showed lower affinity for atrazine than R12.23 and some non-specific binding to magnetic beads. R12.23, however, showed high affinity and specific binding for atrazine, and was therefore chosen for further binding analysis. Mfold predicted that this sequence had a Gibbs energy value of −11.23 kcal·mol^−1^ and therefore a high degree of stability (Figure 3). This sequence was not evident in any rounds prior to Round 12.

**Table 1:** Systematic Evolution of Ligands by Exponential Enrichment (SELEX) scheme for atrazine molecular recognition element (MRE) selection.

Round	Positive Selection	Negative Selection
1	Immobilized Target (IT) 25 h	Immobilization Substrate (IS) 23 h
2	IT 23 h	-
3	IT 18 h	IS 18 h
4	IT 12 h	-
5	IT 10 h	IS 16 h
6	IT w/methanol buffer 12 h	-
7	IT 10 h, Competitive Elution w/1 mM Atrazine (CE) 5 min	-
8	IT 2 h, CE 2 min	IT 2 h, CE w/propanil, 2,4-D acid malathion, 5 min
9	IT 5 min, CE immediate	IT 5 min, CE w/DACT 5 min
10	IT 1 min, CE immediate	IT 5 min, CE w/Simazine 5 min
11	IT immediate, CE immediate	IS 18 h
12	IT immediate, CE w/100 µM atrazine	-

*In vitro* selection process for obtaining atrazine-specific MRE. Immobilization target (IT) is desethyl atrazine bound to magnet beads. Immobilization substrate (IS) is streptavidin-coated magnetic beads plus blocked biotin reagent. Competetive elution (CE) is removal of bound ssDNA from target-coated magnetic beads by free pesticide in solution. Times listed are incubation times in hours (h) or minutes (min).

**Table 2:** Sequence families obtained after 12 rounds of SELEX.

R12.28	TGTACCGTCTGAGCGATTCGTACCATTAGTGGGTGCTCCTTACCTGATGGTCATCTAGCCAGTCAGTGTTAAGGAGTGC
R12.57	TGTACCGTCTGAGCGATTCGTAC 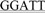 GGGTTTGCACTTTACCTGCGGTGCATCGCAGCCAGTCAGTGTTAAGGAGTGC
R12.59	TGTACCGTCTGAGCGATTCGTAC 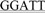 GGGTTTGCACTTTACCTGCGGTGCATCGCAGCCAGTCAGTGTTAAGGAGTGC
R12.23	TGTACCGTCTGAGCGATTCGTACGAACGGCTTTGTACTGTTTGCACTGGC 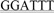 AGCCAGTCAGTGTTAAGGAGTGC
R12.31	TGTACCGTCTGAGCGATTCGTACTAGGAATCCAGCGGAAAAGGC 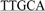 TTATCATGAGCCAGTCAGTGTTAAGGAGTGC
R12.49	TGTACCGTCTGAGCGATTCGTACAGTTCTAGTAGGCGTTAGCATAAATGTTTG 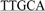 GCCAGTCAGTGTTAAGGAGTGC
R12.23	TGTACCGTCTGAGCGATTCGTACGAACGGCTTTGTACTGT 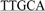 CTGGCGGATTTAGCCAGTCAGTGTTAAGGAGTGC
R12.24	TGTACCGTCTGAGCGATTCGTACAAGAGACTCGGCTTTTGTAATC 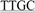 GGTTTTGGAGCCATTCATTGTTAAGGATTGC
R12.26	TGTACCGTCTGAGCGATTCGTACGGCTAGAGTTGTTATGTTTCGATGGTCATCTGCAAGCCAGTCAGTGTTAAGGAGTGC
R12.34	TGTACCGTCTGAGCGATTCGTACCGGTTCTTGAGCGGCTGAATAGTATTTTTC 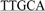 GCCAGTCAGTGTTAAGGAGTGC
R12.10	TGTACCGTCTGAGCGATTCGTACTCATTTGTGGCATTTGAGCGTCAGGGGTAAAGGTAGCCAGTCAGTGTTAAGGAGTGC
R12.23	TGTACCGTCTGAGCGATTCGTACGAACGGCTTTGTACTGTTTGCACTGGCGGATTTAGCCAGTCAGTGTTAAGGAGTGC
R12.24	TGTACCGTCTGAGCGATTCGTACAAGAGACTCGGCTTTTGTAATCTTGCGGTTTTGGAGCCATTCATTGTTAAGGATTGC
R12.23	TGTACCGTCTGAGCGATTCGTACGAACGGCTTTGTACTGTTTGCACTGGCGGATTTAGCCAGTCAGTGTTAAGGAGTGC
R12.65	TACCGTCTGAGCGATTCGTACCATCAGTAGAGTGCGCACTGTAGTAGATGGTCTTAGCCAGTCAGTGTTAAGGAGTGC
R12.3	TGTACCGTCTGAGCGATTCGTACAGTAAGGCACTGGGGCCTTATGCTGTGAGGGATAAGCCAGTCAGTGTTAAGGAGTGC
R12.33	TGTACCGTCTGAGCGATTCGTACTAAGCGACAGAGCACTGTTGCTGTTACA**GTATC**CAGCCAGTCAGTGTTAAGGAGTGC
R12.6	TGTACCGTCTGAGCGATTCGTACTGGCGTAGGGTC**GTATC**TCTTTAAGTGCTGTACTAGCCAGTCAGTGTTAAGGAGTGC
R12.37	TGTACCGTCTGAGCGATTCGTACTCGTCTACATTTACTGGGTTGATGATGA**GTAT**TCAGCCAGTCAGTGTTAAGGAGTGC

Representative sequence families following Round 12 of SELEX. Families are separated by a space with common sequences underlined and sub-families double-underlined.

### 2.2. Affinity and Specificity of Atrazine-Specific MRE

**Figure 4:**
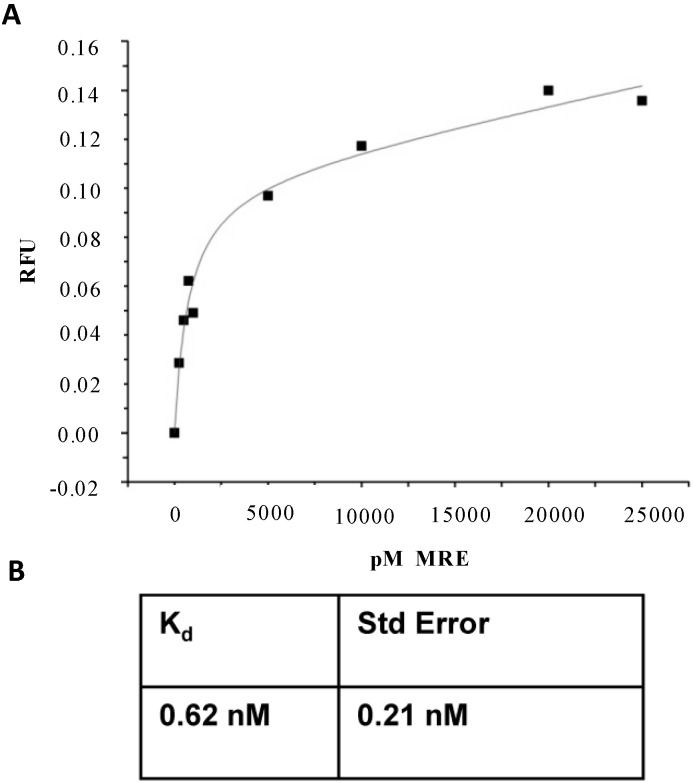
Fluorescent equilibrium binding assays of R12.23. Data represent K_d_ of R12.23 for three saturation binding curves. (**A**) Representative saturation binding curve of R12.23 with nonlinear regression best fit; (**B**) Equilibrium dissociation constant with standard error of R12.23 binding assays.

Fluorescent saturation binding assays were performed to determine the affinity of MREs for atrazine. Concentrations of MRE in the high pM to low nM range were assayed. The equilibrium dissociation constant (K_d_) was 0.62 ± 0.21 nM for R12.23, 5.10 ± 1.32 nM for R12.28, and 20.78 ± 4.74 nM for R12.57 (Figure 4). This bead-based fluorescence saturation binding assay method has previously been used to identify sub-nanomolar MRE affinities [34]. Therefore, R12.23 was chosen as the atrazine MRE to be used in further analysis. This is in the lower range of K_d_ values for MREs targeting small molecules, though affinities this high have been obtained before, and comparable to MREs targeting proteins [35,36]. As noted above, a DNA MRE was identified for atrazine with a K_d_ of 890 nM, 1000 times higher than the MRE selected here [21]. This, combined with the inherent stability of DNA, makes it an excellent candidate for environmental sensing applications. Such high affinity for atrazine is likely due to the multiple rounds of selection with stringent binding conditions, thus validating the stringency of our SELEX scheme.

Fluorescent specificity binding assays were also performed on the atrazine MRE through elution of the MRE bound to target beads when free target was added. The data is presented relative to R12.23 binding to atrazine as has been previously described [37,38,39]. Analysis showed significantly higher binding of R12.23 to atrazine in solution than to four of the five negative targets used in the selection (*p* < 0.05) (Table 3). Binding of the MRE to atrazine was 2.1× greater than DACT, 2.1× greater than simazine, 1.5× greater than propanil, and 1.5× greater than 2-4,D Acid. Binding to atrazine was 1.1× greater than to malathion, but this difference was not significant due to high variability in malathion binding. This is likely because malathion is known to be reactive toward DNA by cleaving it [40,41,42]. This reactivity of malathion may not affect the signal of a field-deployable sensor if it is designed to function based on MRE folding or if it is designed to be dependent on an MRE of a specific size as previously described [43]. It is important to note that R12.23 is selective for atrazine over simazine, as current molecular recognition elements for atrazine are not [19]. Also, the MRE has negligible affinity for desethyl atrazine in solution. This is noteworthy because this atrazine derivative was immobilized on magnetic beads and used for the initial positive rounds of selection. It is clear that stringent competitive elutions selected for an MRE that has high specificity for atrazine in solution compared to the negative targets, further validating the stringency of our negative selections.

**Table 3:** Cross-reactivity data of R12.23 ssDNA MRE.

Eluent	Normalized Average Fluorescence	Standard Deviation	*p*-Value	Selectivity Ratio
Atrazine	1	0.11	-	-
Desethyl Atrazine	−0.054	0.44	0.01	Neg. *
DACT	0.47	0.10	0.002	2.1
Simazine	0.47	0.14	0.003	2.1
Propanil	0.65	0.13	0.01	1.5
2-4,D Acid	0.64	0.11	0.009	1.5
Malathion	0.92	0.46	0.39	1.1
MSB	0.15	0.19	0.001	6.6

For each eluent, normalized average fluorescence in solution is given with standard deviation. The *p*-value is given from a student’s *t*-test performed between the eluent and atrazine eluted fluorescence intensity. The selectivity ratio is the number of times greater binding to atrazine than to the eluent. MSB is methanol selection buffer as described. * Neg. denotes negligible binding of R12.23 to desethyl atrazine and thus a very large Selectivity Ratio.

### 2.3. Atrazine Detection in Environmental Conditions

**Figure 5:**
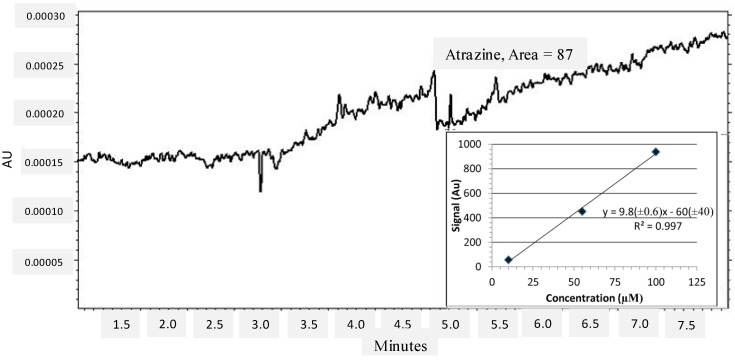
Separation and detection of MRE-bound atrazine from processed river water at an absorbance of 225 nm.

The high affinity and specificity of the atrazine MRE are both important characteristics for its further use in detection applications, such as contaminated ecosystems or individuals. The potential of the atrazine MRE for environmental analysis was evaluated using a 34 base truncated sequence for cost and processing efficiency which spans the stable binding region of the MRE. River water was spiked to contain 40 nM (*i.e.*, 4.0 nmol) atrazine, which is below the level of concern in aquatic ecosystems reported by the EPA as 10 ppb [44,45]. The spiked sample was processed, incubated with immobilized MRE, and captured atrazine was quantified using capillary electrophoresis. The resulting electropherogram (Figure 5) confirms that 37% (1.5 nmol) of the atrazine was recovered from the river water. This confirms the utility of this MRE in environmental sensing applications and future work will compare this MRE to other detection methods.

Nucleic acid MREs have previously been developed and used in environmental biosensing for small molecules [46,47]. It is also likely, as has been previously been reported, that this MRE will be incorporated into a multiplexed sensing platform that detects other atrazine metabolites as well as other pesticides [48]. They have also been used for detection of analytes in serum [32]. In both contexts, it is necessary for the MRE to differentiate between the target and closely-related molecules or other contaminants. The previously-noted atrazine MREs also do not show specificity for atrazine. The atrazine DNA MRE was not selected against simazine, although simazine was used to test specificity. There was no specificity below approximately 2 µM [21]. We have demonstrated in this work that there is specificity for atrazine over simazine and other metabolites and pesticides using as little as 5 nM of the selected ssDNA MRE. The stringency of the SELEX methodology reported herein is thus capable of generating MREs with very high specificity and affinity and the selected R12.23 MRE will be much more useful in environmental sensing applications than other atrazine MREs. Additionally, we have shown the ability of the MRE to detect atrazine in environmental samples at environmentally relevant concentrations. Using such a sensor, identified atrazine-polluted environments or contaminated at-risk individuals can be actively remediated or treated with chemical, physical or biological methods [49,50,51].

## 3. Experimental Section

### 3.1. SELEX for Selection of Atrazine-Specific MREs

In order to select an atrazine-specific MRE, the SELEX process was used (Figure 1). A single stranded DNA (ssDNA) library consisting of 10^15^ different molecules was screened through 12 rounds of selection (Table 1). The ssDNA library, termed RMW.N34, consists of two 23 base constant priming regions for polymerase chain reaction (PCR) amplification flanking a 34 base random region (synthesized by Eurofins MWG Operon; Huntsville, AL, USA). From this library, molecules that have a sequence and tertiary structure that specifically binds to atrazine were selected. Molecules in the library which bound to closely-related structures and other pesticides were removed (Figure 2).

An atrazine derivative, desethyl atrazine (Figure 2B) (Fluka Analytical; St Louis, MO, USA) was covalently biotinylated by Sulfo-NHS-LC-Biotin (Pierce; Rockford, IL, USA) per manufacturer’s instructions (Figure 1A). Biotinylated desethyl atrazine was then incubated with streptavidin-coated magnetic beads (New England Biolabs; Ipswich, MA, USA) to immobilize the selection target. Unbound desethyl atrazine was washed to leave only biotinylated desethyl atrazine bound to magnetic beads (immobilized target).

For positive immobilized target selection, the ssDNA library was incubated with 50 μL immobilized target in 500 µL of buffer consisting of 100 mM sodium chloride, 20 mM Tris-HCl, and 2 mM magnesium chloride (1X selection buffer; SB). After an incubation period with rotation at room temperature, the immobilized target was separated from the solution by magnet, washed three times with 1 mL SB, and resuspended in SB. This suspension served as a template for PCR amplification of bound ssDNA. PCR conditions were as follows: enriched ssDNA library, 400 nM forward and biotinylated reverse RMW.N34 primers (Eurofins MWG Operon; Huntsville, AL, USA) (forward: 5'-TGTACCGTCTGAGCGATTCGTAC-3', biotinylated reverse: 5'-Biotin-GCACTCCTTAACACTGACTGGCT-3'), 250 µM deoxynucleotide triphosphates, 1× GoTaq Reaction Buffer (Promega; Madison, WI, USA), 3.5 units *Taq*, and pure water. Reaction conditions were: initial denaturation of 95 °C for 5 min; cycling at 95 °C for 1 min, 63 °C for 45 s, and 72 °C for 1 minute; and final extension of 72 °C for 7 min. Large-scale amplification of 4 and 2 mL was done preceding positive and negative selection rounds, respectively. This selection scheme for the desethyl atrazine target occurred for Rounds 1–5 of selection, each time with a shorter incubation period.

Amplified dsDNA was then purified via the GFX PCR purification kit (GE Healthcare; Piscataway, NJ, USA). Eluted dsDNA from the column was incubated with streptavidin agarose resin (Pierce; Rockford, IL, USA). Having used a biotinylated primer for the reverse strand amplification allowed for binding of the amplified dsDNA to the agarose resin. This was placed into a flow-through column and washed with 5 volumes of 1× PBS. Five volumes of 1 M sodium hydroxide were then used to dissociate the DNA strands, eluting the forward strand ssDNA from the column. This solution was neutralized with 0.1 volume 3 M sodium acetate (pH 5.2), to which 2.5 volumes of cold 100% ethanol were added. Glycogen at 10 μg·mL^−1^ was added as a co-precipitate for ethanol precipitation, and the solution was frozen. After freezing, this was centrifuged at 13,000× *g* for 1 h, washed with 70% ethanol, and centrifuged again for 15 min. The supernatant was then decanted and the ssDNA pellet dried under a vacuum. The purified ssDNA pellet was resuspended in SB and subjected to another selection incubation.

Multiple, strong negative selections were performed to ensure the ssDNA MRE is specific for atrazine. Sulfo-NHS-LC-Biotin (Pierce; Rockford, IL, USA) was blocked with Tris-HCl. Then the blocked biotin reagent was incubated with streptavidin coated magnetic beads and washed with SB (immobilization substrate). 50 µL of blocked biotin reagent immobilized onto magnetic beads was incubated with purified ssDNA from the positive selection in a 100 µL volume. After an incubation period, the supernatant was removed and beads were washed three times with 25, 25, and 50 µL SB, with removed supernatant and washes serving as a PCR template. Amplified dsDNA was then prepared for another selection round as outlined above. Negative selections done in this manner occurred in Rounds 1, 3, 5, and 11.

Round 6 of selection was done as stated above, however the selection buffer consisted of 10% methanol in 1× selection buffer (methanol selection buffer; MSB) to ensure ssDNA binding in ensuing selection rounds. For Rounds 7–11 of positive selection, the purified ssDNA library was incubated with immobilized target as outlined above. However, after three washes, 100 μL of 1 mM atrazine in MSB was incubated with the beads for decreasing time periods. The ssDNA in the solution phase was separated from the immobilized target and served as a template for PCR amplification. Preparation of the library for the ensuing round of selection was done as noted above.

More negative selections were performed on closely-related and environmentally-local molecules. The Round 8 negative selection was done as described above, however ssDNA was competitively eluted with the pesticides propanil, 2,4-D acid, and malathion (Figure 2E–G). The ssDNA bound to the immobilized target was separated from solution and resuspended, serving as the template for PCR amplification. Rounds 9 and 10 of negative selection were done in the same manner, with DACT and simazine (Figure 2C,D) serving as targets for competitive elution, respectively.

The final round of positive selection, Round 12, was done similar to the competitive elutions above. However, bound ssDNA was eluted with 100 μL of 100 μM atrazine in MSB. This eluted ssDNA served as a template for PCR amplification.

### 3.2. Sequencing of Atrazine-Specific MREs

Every third round of selection, the ssDNA library was analyzed for consensus binding sequences by DNA sequencing. The PCR-amplified (using non-biotinylated primers) library was ligated into the pCRII vector (Invitrogen, Carlsbad, CA, USA) and cloned into competent bacteria per manufacturer’s instructions. The plasmid was then extracted using the AxyPrep Plasmid Prep Kit (Axygen, Union City, CA, USA) and sent for sequencing (Eurofins MWG Operon, Huntsville, AL, USA) using the M13R primer complementary to a region upstream of the PCR insert in the pCRII vector. Analyses were done on 30–50 random sequences for each round to identify consensus sequence binding families (Table 2).

### 3.3. Atrazine MRE Binding Assays

From the Round 12 sequences, three sequences were chosen for further characterization. The predicted tertiary structures were obtained using the Mfold DNA web server at 25 °C with buffer salt conditions [33]. R12.23, R12.28, and R12.57 were chosen and synthesized by Eurofins MWG Operon with an AlexaFluor 488 label for use in binding studies.

Fluorescence saturation bead-based binding assays were performed as previously described [34,52,53] Concentrations of 0, 0.25, 0.5, 0.75, 1, 5, 10, 15, 20, and 25 nM MRE were used in saturation binding studies. In a 200 µL total volume of MSB, 10 µL of immobilized target was incubated with each concentration for five minutes. Unbound MRE was removed and each incubation was washed five times with 200 µL MSB, then the immobilized target was resuspended in 200 µL MSB and heated to 95 °C for 10 min to denature and elute bound MRE. Eluted ssDNA in MSB was placed in a 96-well microplate and measured in a Synergy 2 microplate reader equipped with a tungsten halogen lamp with excitation wavelength of 490 nm and an emission filter at 520 nm using Gen5 1.06 software (Biotek US; Winooski, VT, USA). All fluorescence readings on the plate were normalized to 100 μL of a 1 nM solution in SB of the fluorescent MRE and the no ssDNA incubation. For each concentration set, the same was done with washed streptavidin-coated magnetic beads to ensure binding over background to the beads. Each set of incubations were performed in triplicate. To determine the dissociation constant of the MREs, data were analyzed with Origin 8 (OriginLab Corporation; Northampton, MA, USA) using nonlinear regression analysis and fit with the equation, Y = ((B_max_ × X)/(K_d_ + X)) + NS × X, where B_max_ is the maximum binding, K_d_ is the dissociation constant, and NS is nonspecific binding.

To determine binding of the selected MRE, R12.23, to negative targets used in the selection, 5 nM fluorescent ssDNA was used. For each eluent used, in a 200 µL total volume of MSB, 10 µL of immobilized target was incubated with R12.23 for five minutes. The magnetic beads were washed five times with 200 µL MSB. Then, each incubation was resuspended in 200 µL of 100 µM in MSB of the following in MSB: atrazine, desethyl atrazine, DACT, simazine, propanil, 2-4,D Acid, malathion, and MSB only. An incubation with no R12.23 present and with atrazine as the eluent served as normalization controls. Each eluent was incubated with the immobilized target for five minutes, and the solution was removed from the beads. This solution was placed in a 96-well plate and measured in a fluorescence plate reader. Data were normalized to an internal fluorescent standard and then the no-R12.23 control was subtracted. Each set of specific binding studies were performed in triplicate. Data were averaged and standard deviations calculated, with values presented relative to average atrazine binding as previously described [37,38,39]. For each eluent, a student's t-test was performed to determine statistical differences in the means.

### 3.4. Atrazine Detection in River Water

#### 3.4.1. MRE Bead Immobilization

A 360 µL sample of amine-terminated silica-coated magnetic beads provided at a concentration of 30 mg/mL (#FA-101, Bioclone Inc., San Diego, CA, USA) were rinsed with 3 mL (1 mL × 3 times) of 10 mM phosphate buffer at pH 8. The supernatant was removed and replaced with 360 µL of 30 mM (4-4(4-maleimidophenyl) butyric acid *N*-hydroxy succinimide ester) (Sigma Aldrich, Saint Louis, MO, USA) crosslinker dissolved in 50 µL dimethylsulfoxide and 310 µL phosphate buffer. The solution was incubated for 1 hour at room temperature on a rocker. The beads were rinsed with 3 mL (1 mL × 3) Tris (10 mM)–EDTA (1 mM) buffer. Thiol-modified MRE (15 nmoles) (5-/5ThioMC6-D/TACTGTTTGCACTGGCGGATTTAGCCAGTCAGTG-3) (Integrated DNA Technology, Coralville, IA, USA) and 47 mM (tris-(2-carboxyethyl)phosphine, hydrochloride (Life Technologies, Grand Island, NY, USA) were added to the beads in Tris-EDTA buffer. This 34-base truncation of the selected MRE spanning the stable binding complex was used due to cost of preparation and efficiency of separation. The solution was incubated 180 min at room temperature on a rocker. The sample was rinsed with SB and stored at 4 °C.

#### 3.4.2. Sample Processing

River water was collected from the Potomac River at GPS coordinates 39°00'00.04"N 79°03'05.78"W. River water (0.1 L) was decanted to exclude visible debris and spiked to contain 40 nM atrazine. The spiked water sample was processed through a C-18 cartridge (DSC18 #52601-U, Supelco, Bellefonte, PA, USA). Prior to use, cartridges were rinsed with 5 mL of HPLC grade methanol and 5 mL deionized water. Water samples were loaded onto cartridges, rinsed with 2 mL of deionized water, and eluted in 1 mL methanol. The methanol fraction was evaporated to dryness at ambient temperature using a SpeedVac concentrator (Thermo Scientific, Waltham, MA, USA) in approximately 90 min. The dried sample was reconstituted in 100 µL of SB. The recovery of atrazine with this processing protocol was 130% when evaluated with 100 mL of river water. Beads with immobilized MRE were added to the reconstituted sample after being heated to 95 °C and cooled to room temperature. The sample was then incubated with beads for 60 min. The supernatant was removed by magnetic separation. Deionized water (100 µL) was then added to the beads and heated to 95 °C for 10 min to elute bound atrazine. The supernatant was removed by magnetic separation and the bound atrazine was quantified using capillary electrophoresis with UV visible absorbance detection.

#### 3.4.3. Atrazine Quantification

Capillary electrophoresis was used to separate, detect, and quantify atrazine. Separations were performed with a Beckman Coulter P/ACE MDQ (Beckman Coulter, Fullerton, CA, USA) equipped with a UV-visible detector, which was operated at 225 nm. Separations were accomplished using a 25 µm inner diameter, 360 µm outer diameter, 60 cm long fused silica separation capillary (Polymicro Technologies, LLC, Phoenix, AZ, USA). The detection window was positioned 50 cm from the anodic reservoir. The capillary was conditioned daily with the following flushes at 172 kPa (25 psi): 30 min 1 N sodium hydroxide, 15 min water, 15 min methanol, 15 min water, 10 min with background electrolyte used for the capillary electrophoresis separation. The background electrolyte is comprised of 30 mM SDS, 30 mM phosphate buffered at pH 10. Between runs the capillary was flushed at 172 kPa (25 psi) for 3 min with the running electrolyte. The capillary cartridge was maintained at 25 °C. Separations were performed at 24 kV under normal polarity (anode at the point of injection, cathode at the capillary outlet). The background electrolyte was made fresh every day prior to use. The eluted atrazine sample was introduced using pressure 7 kPa (1 psi) for 6 s. Data collection and analysis were performed using 32 Karat Software version 5.0 (Beckman Coulter) and theoretical plates and peak width at 50% height were calculated with USP criterion software enabled.

## 4. Conclusions

A stringent SELEX methodology is demonstrated to isolate a molecular recognition element with high affinity and specificity for the herbicide atrazine. The MRE binds with a sub-nanomolar equilibrium dissociation constant and is selective for atrazine over several metabolites and other pesticides used as negative targets in the selection. Additionally, we were able to detect atrazine in spiked river water samples at environmentally relevant concentrations. The results validate our SELEX process while identifying a new tool for atrazine detection in environmental and biological mediums.
